# Sludge water: a potential pathway for the spread of antibiotic resistance and pathogenic bacteria from hospitals to the environment

**DOI:** 10.3389/fmicb.2025.1492128

**Published:** 2025-02-12

**Authors:** Bingxuan Zhao, Rui Zhang, Baolin Jin, Zuozhou Yu, Weicheng Wen, Tong Zhao, Yue Quan, Jingya Zhou

**Affiliations:** ^1^Department of Environmental Science, Yanbian University, Yanji, China; ^2^Department of Clinical Medicine, Yanbian University, Yanji, China; ^3^Agricultural College, Yanbian University, Yanji, China; ^4^Department of Biotechnology, Yanbian University, Yanji, China

**Keywords:** antibiotic resistance, antibiotic resistance genes, human pathogenic bacteria, hospital wastewater, public health

## Abstract

Hospitals play an important role in the spread of antibiotic resistance genes (ARGs) and antimicrobial resistance (AMR). The ARGs present in hospital wastewater tend to accumulate in activated sludge, with different ARGs exhibiting varying migration rates. As a result, sludge water produced during the activated sludge treatment process may be a significant source of ARGs entering the environment. Despite this, research into the behavior of ARGs during sludge concentration and dewatering remains limited. This study hypothesizes that ARGs might exhibit new behaviors in sludge water during sludge concentration. Using metagenomic analysis, we explored the distribution and migration risks of ARGs and human pathogenic bacteria (HPB) in sludge water, comparing them with those in hospital wastewater. The findings reveal a strong correlation between ARGs in sludge water and hospital wastewater, with subtypes such as *arlR*, *efpA*, and *tetR* showing higher abundance in sludge water. Although the horizontal gene transfer potential of ARGs is greater in hospital wastewater than in sludge water, the resistance mechanisms and migration pathways are similar even when their HPB host associations differ. ARGs in both environments are primarily transmitted through coexisting mobile genetic elements (MGEs). This suggests that sludge water serves as a critical route for the release of hospital-derived ARGs into the environment, posing potential threats to public health and ecological safety.

## 1 Introduction

AMR is considered one of the major global threats to public health and environmental safety, causing hundreds of thousands of deaths annually due to bacterial infections and affecting human life expectancy worldwide (Pires et al., 2017). Antibiotic overuse has significantly driven the evolution of ARGs and antibiotic-resistant bacteria (ARB) (Zainab et al., 2020). The prevalence of ARGs in hospital wastewater has attracted global attention, prompting researchers to assess resistance levels and risks through water sample analysis (Perry et al., 2021). ARGs can spread between bacteria via horizontal gene transfer (HGT), with MGEs playing a critical role in this process (Chen et al., 2021). Marion Hutinel et al. identified two ARG subtypes, *sul4* and *gar*, in Swedish hospital wastewater for the first time, which had never been identified in Sweden before, highlighting the significant transmission risk of ARGs (Hutinel et al., 2022). In developing nations, hospital wastewater is a major contributor of HPB and ARGs in the environment (Khan et al., 2019), threatening aquatic ecosystems and increasing public health and safety risks (Kaliakatsos et al., 2024).

The biological treatment of hospital wastewater produces activated sludge rich in ARGs and ARB. The complex composition of sludge and microbial interactions contribute to the uncertainty of ARG and ARB transmission mechanisms (Yin et al., 2024). ARGs demonstrate variable spatial distributions in sludge flocs, with differing migration rates observed among ARGs in sewage sludge (He et al., 2019). Activated sludge contains water in various physical states, including free water, interstitial water, surface water, and bound water associated with sludge solids (Vaxelaire and Cézac, 2004). Disruption of sludge cells alters the spatial distribution of water within the sludge (Erdincler and Vesilind, 2003). Therefore, this study speculates that ARGs in sludge water generated during sludge concentration may display novel behavioral characteristics. The factors driving the distribution and migration of ARGs in sludge water are yet to be identified, highlighting the need to clarify the regulatory role of MGEs in ARG migration from hospital wastewater to sludge water. This is essential to advance the One Health framework and combat AMR effectively.

Wastewater-based monitoring that does not rely on cultivation provides an effective approach to detect AMR (Majlander et al., 2021), with ARGs and ARB in hospital wastewater serving as indicators of environmental risks and predictors of clinical AMR (Cai et al., 2021). Previous research has largely emphasized ARGs in the wastewater treatment process and sludge flocs, overlooking their dynamic behavior during sludge dewatering. Thus, it remains crucial to investigate ARG transmission pathways to mitigate the environmental risks associated with AMR. This study investigates ARG mobility and abundance characteristics, delves into their transmission mechanisms, and assesses the prevalence of ARGs, MGEs, and ARB in hospital wastewater and sludge water, emphasizing ARG-hosting HPB. The key drivers facilitating ARG diffusion from hospital wastewater to sludge water were identified, alongside an analysis of ARG characteristics in these two environments. This study aims to reveal the migration risks of ARGs in hospital wastewater and sludge water, offering theoretical and scientific foundations to mitigate the worsening AMR threat.

## 2 Methods

### 2.1 Sample collection and processing

**Figure 1:**
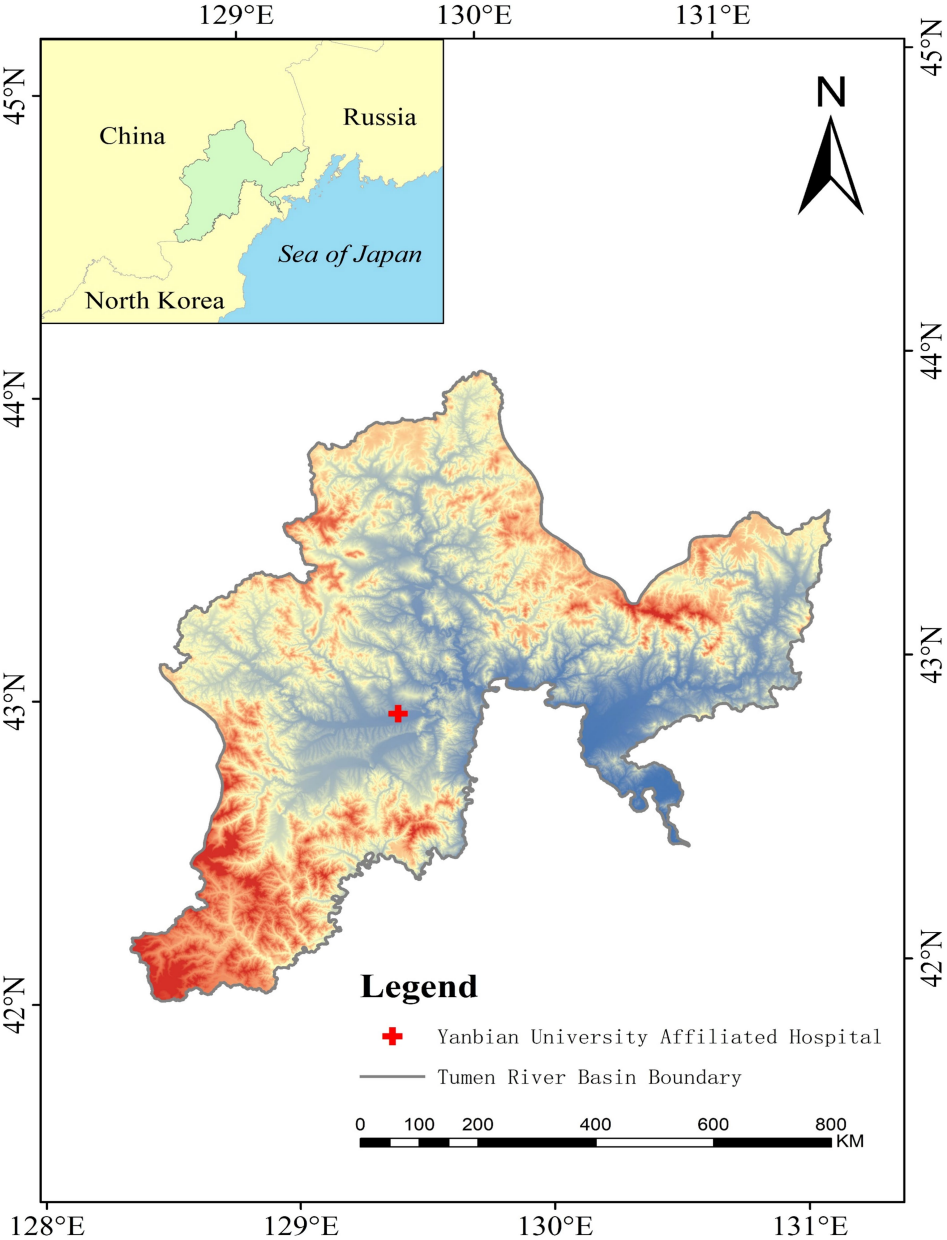
The map of Yanbian hospital.

Situated in northeastern China, the Tumen River Basin houses the Yanbian Hospital, the only third-grade modern general hospital in the region with over 1,500 beds, serving as the central hospital (Figure 1). Given its use of the activated sludge process for wastewater treatment, this hospital presents a critical case for investigating ARGs in hospital wastewater and sludge water. In addition, the Tumen River, positioned at the intersection of China, North Korea, and Russia, is geographically unique and includes transboundary water pollution. Research on water pollution in this region is important for advancing international water resource management and conservation efforts.

In this study, sterile equipment was used to collect wastewater and sludge water samples from Yanbian Hospital. Samples were taken from three different locations and then pooled in 1 L sterile polyethylene bottles. Each collection was performed in triplicate for biological replicates, with sample records documented (Table 1). Immediately after collection, the samples were placed on dry ice and transported to the laboratory for processing. A 0.2-μm filter membrane was used for filtration with a vacuum filtration system to capture material on the membrane. The filter membrane was then collected for subsequent DNA extraction to ensure accurate downstream analysis.

**Table 1:** Sample name records.

Group	Name	Information	Sampling time	Sampling location
Wastewater	WW_1	Hospital wastewater	2023.6.10	The inlet of the hospital wastewater
Wastewater	WW_2	Hospital wastewater	2023.6.10	The inlet of the hospital wastewater
Wastewater	WW_3	Hospital wastewater	2023.6.10	The inlet of the hospital wastewater
Sludge water	SW_1	Sludge water	2023.6.10	The outlet of the sludge concentration device
Sludge water	SW_2	Sludge water	2023.6.10	The outlet of the sludge concentration device
Sludge water	SW_3	Sludge water	2023.6.10	The outlet of the sludge concentration device

### 2.2 Metagenomic sequencing

The total DNA of water samples was extracted using the Yuehua (Meiji, China) kit, and then the DNA was subjected to ultrasonic fragmentation, and the DNA library was constructed according to the standard process provided by Illumina. Metagenomic sequencing was performed using the Illumina NovaSeq6000 (Illumina, United States) sequencing platform (Shanghai Meiji Biomedical Technology Co., Ltd.). Data quality control (fastp) (Chen et al., 2018) and assembly splicing (MEGAHIT) (Hocquet et al., 2016; Li et al., 2016). Then, Open reading frames (ORFs) from each assembled contigs were predicted using Prodigal (Martínez et al., 2015). The predicted ORFs with a length ≥ 100 bp were retained and translated into amino acid sequences.

### 2.3 Annotation of ARG-like ORFs

Gene annotation for ARGs includes information on the ARG subtype, ARG type, and resistance mechanism. The ARG subtype refers to the gene name of the ARGs (e.g., *tetW* and *sul1*), the ARG type indicates the antibiotic resistance type of ARGs (such as Tetracycline and Sulfonamide), and the resistance mechanism provides information on the specific mechanism of ARGs.

The annotation of ARG-like ORFs was accomplished using DeepARG (Arango-Argoty et al., 2018) (V 1.0.2, DeepARG-LS Model) with default parameters. The abundance (coverage, ×/Gb) of ARGs in each sample was calculated as follows (Ma et al., 2016; Xiong et al., 2018; Zhao et al., 2020) (Equation 1):


(1) $$\begin{array}{l} \mathrm{Abundance}\left(\mathrm{coverage},\times /Gb\right)\\ =\overset{n}{\underset{1}{\sum }}\frac{N_{\mathrm{mapped}\;\mathrm{reads}}\times L_{\mathrm{reads}}/L_{ARG-\mathrm{like}\;\mathrm{ORF}}}{S} \end{array}$$

Where $N_{\mathrm{mapped}\;\mathrm{reads}}$ is the number of the reads mapped to ARG-like ORFs; $L_{\mathrm{reads}}$ is the sequence length of the Illumina reads (150 bp); $L_{ARG-\mathrm{like}\;\mathrm{ORF}}$ is the sequence length of target ARG-like ORFs (bp); *n* is the number of the ARG-like ORFs belonging to the same ARG type, and S is the size of the clean data set (Gb).

### 2.4 Taxonomy annotation of ARG-carrying contigs

The abundance (coverage, ×/Gb) of each ARG-carrying Contigs (ARCs) was calculated through (Equation 1). The ORFs of ARCs were searched against the NCBI non-redundant (NR) protein database (nr_20200306) using DIAMOND (Buchfink et al., 2015) (blastp) with an e-value ≤1e-5. If more than 50% of the ORFs on an ARC were assigned to the same taxonomy rank (domain/ kingdom/ phylum/ class/ order/ family/ genus), then the ARC was assigned to the taxon, and the taxon was identified as the potential host of ARGs (Ma et al., 2017). Moreover, based on the taxonomy results (species level) of ARCs, the ARCs were compared with the established HPB database (Yi et al., 2022) to identify HPB hosts of ARGs. The abundance (coverage, ×/Gb) of each ARG host was calculated with the abundance of ARCs assigned to different taxa.

### 2.5 Horizontal gene transfer analysis

To explore the potential HGT of ARGs among microbes, PlasFlow (V 1.1) (Krawczyk et al., 2018) with default parameters was used to predict the genetic location (plasmid or chromosomal) of ARCs. Meanwhile, to predict the MGEs co-occurrence with ARGs on contigs, the ORFs of ARCs were searched against the MGEs90^1^ (Arango-Argoty et al., 2018) using BLASTP with an e-value ≤1e−5, an identity ≥80% and a query coverage ≥70%. The abundance (coverage, ×/Gb) of MGEs was calculated using Equation 1.

### 2.6 Statistical analysis

Statistical analysis was performed on the metagenomic sequencing results using the Majorbio Cloud Platform.^2^ The Wilcoxon rank-sum test was used to evaluate the significant differences in the proportion of ARCs carrying multiple ARGs and the total abundance of ARGs between different samples. Based on the abundance of ARGs in the sample, log10 was used to standardize the abundance, and the heatmap was visualized to obtain complete information on the antibiotic resistance group in hospital wastewater.

In addition, based on the species annotation of ARCs, the direct relationship between ARGs and their hosts in each group can be constructed. Using R (version 4.0.5), the co-occurrence relationship between ARGs (ARG types) and hosts in different groups was intuitively analyzed. LEfSe analysis was performed on functional genes and hosts with significant differences (*p* < 0.05) between groups using Python, and LDA scores were visualized through a histogram.

The number of ARG types located in plasmid, chromosomes, and unclassified sequences across all samples was counted, and the distribution of ARGs in different genetic locations was visualized. Linear regression analysis was performed using the Bray–Curtis distance matrix from the Mantel results as input to examine the correlation between MGEs (abundance of MGE subtypes), ARGs (abundance of ARG subtypes), hosts (host abundance at the genus level), and HPB (HPB abundance at the species level). In the results, “Mantel_r” represents the correlation *r* value of Mantel test results, the *p*-value is the significance test *p*-value of regression analysis, and the *p*-value <0.05 represents significance.

## 3 Results

### 3.1 Abundance distribution, characteristics, and differences of ARGs

**Figure 2:**
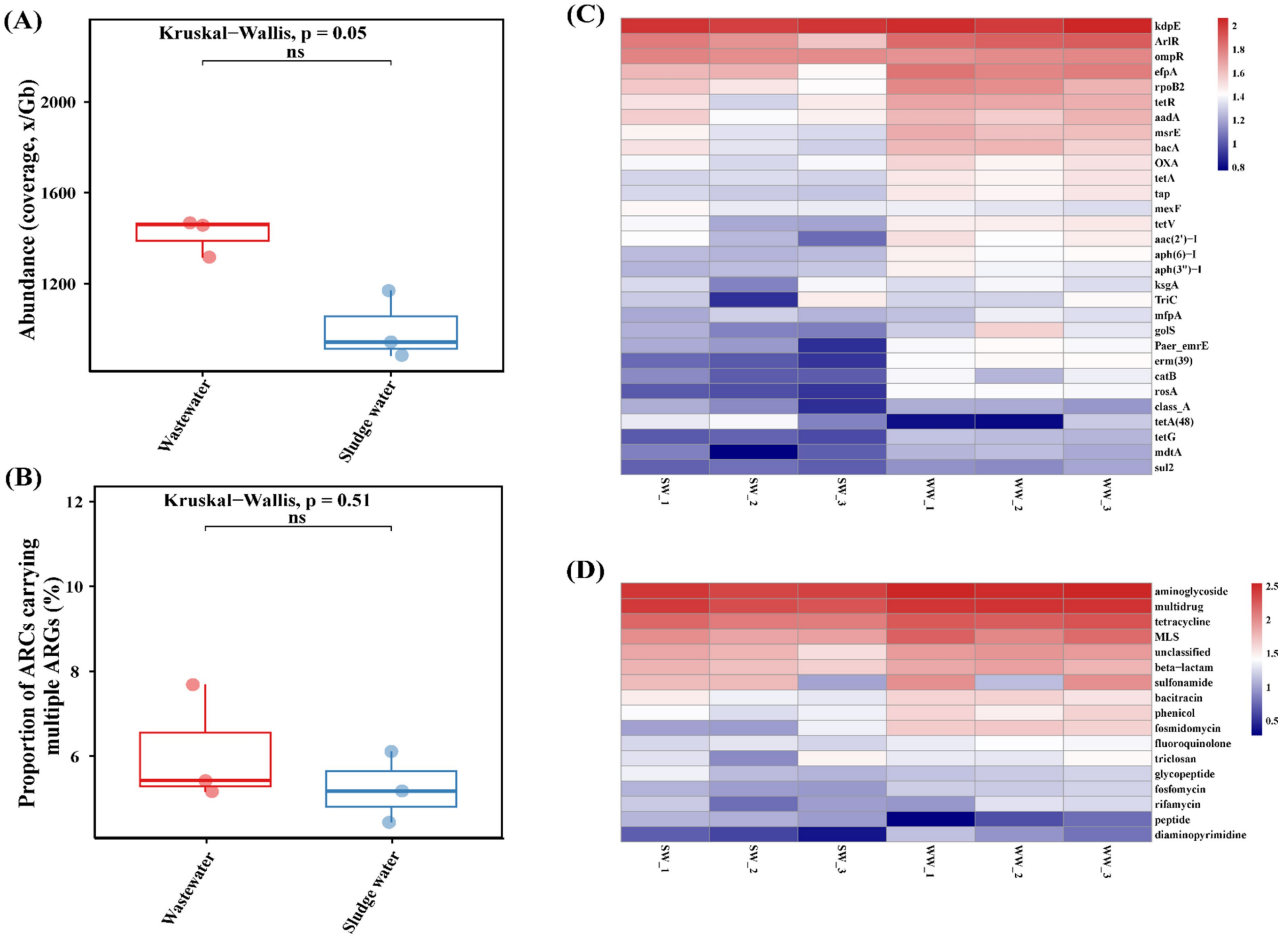
**(A)** Proportion of number of ARCs carrying ≥2 ARGs. **(B)** Total abundance of ARGs. **(C)** ARG subtypes abundance. **(D)** ARG types abundance.

As shown in Figure 2A, the annotation results indicated that the total abundance of ARGs in hospital wastewater samples was higher than that in the sludge water samples. At the same time, as shown in Figure 2B, the proportion of ARCs carrying multiple ARGs in hospital wastewater was also slightly higher than that in sludge water, but there was no significant difference between the two groups (*p* = 0.51).

The ARG subtypes and ARG type of the abundance level top 30 were visualized using a heatmap, and the results are shown in Figures 2C,D. High abundance of aminoglycoside ARGs (*kdpE*, *aac(2′)-I*, *aadA*, etc.), multi-drug resistant ARGs (*rpoB2*, *ompR*, *efpA*, etc.), tetracycline ARGs (*tetR*, *tetA*, *tetV*, etc.), macrolide ARGs (*erm(39)*, *msrE*, *mefA*, etc.), *β*-lactam ARGs (*OXA*, *SHV*, *TEM*, etc.), sulfonamide ARGs (*sul1* and *sul2*) were identified in hospital wastewater and sludge water samples. However, the ARG subtype abundance of the top 30 in sludge water was slightly higher than that in hospital wastewater.

**Figure 3:**
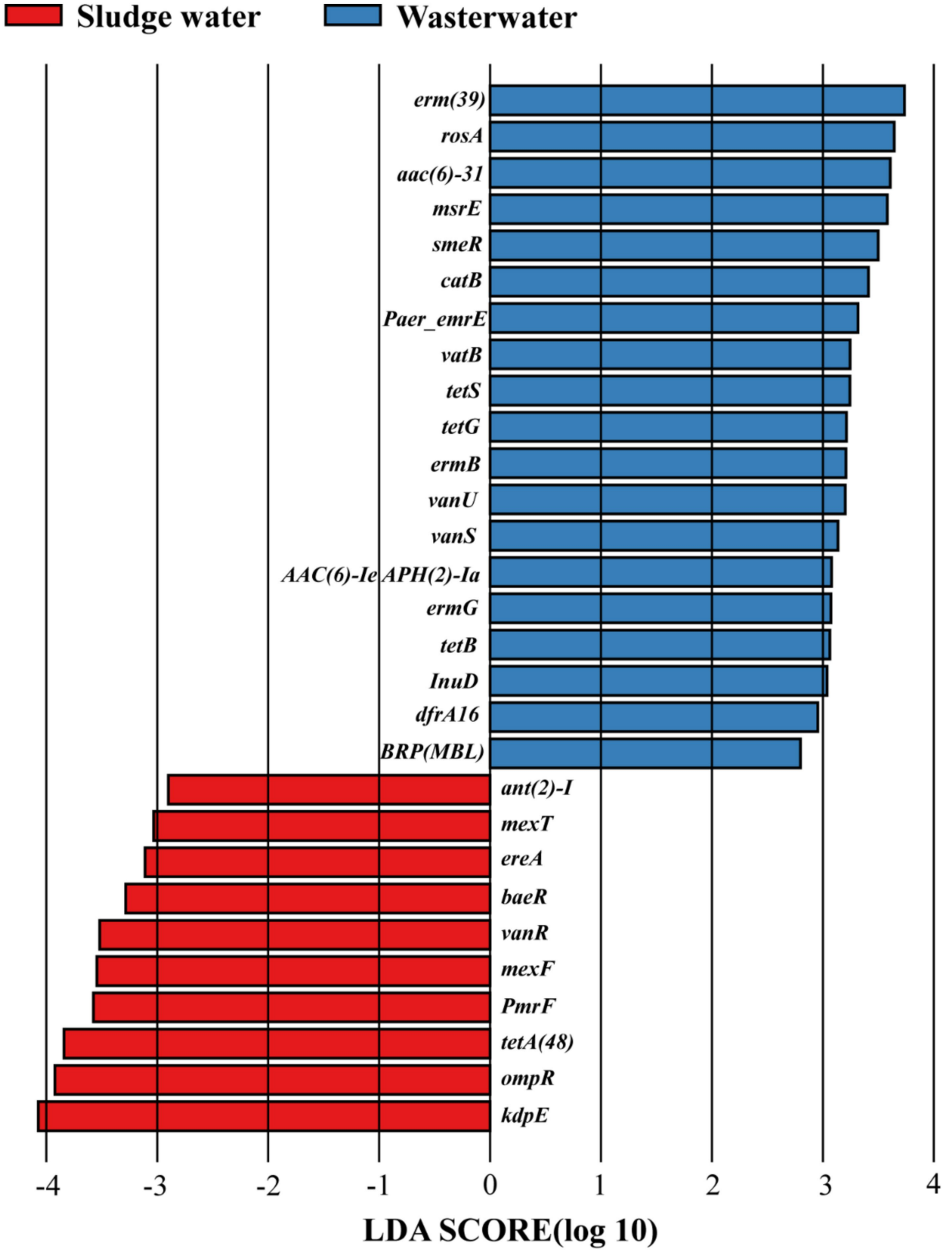
LDA discriminant bar chart.

As shown in Figure 3, there are 29 differentially expressed genes with LDA > 2 in hospital wastewater and sludge water. The larger the LDA value, the greater the influence of the difference. The results showed that in hospital wastewater and sludge water, *kdpE* and *erm(39)* had the greatest influence on the difference in the abundance distribution of ARG subtypes.

### 3.2 Host community characteristics

**Figure 4:**
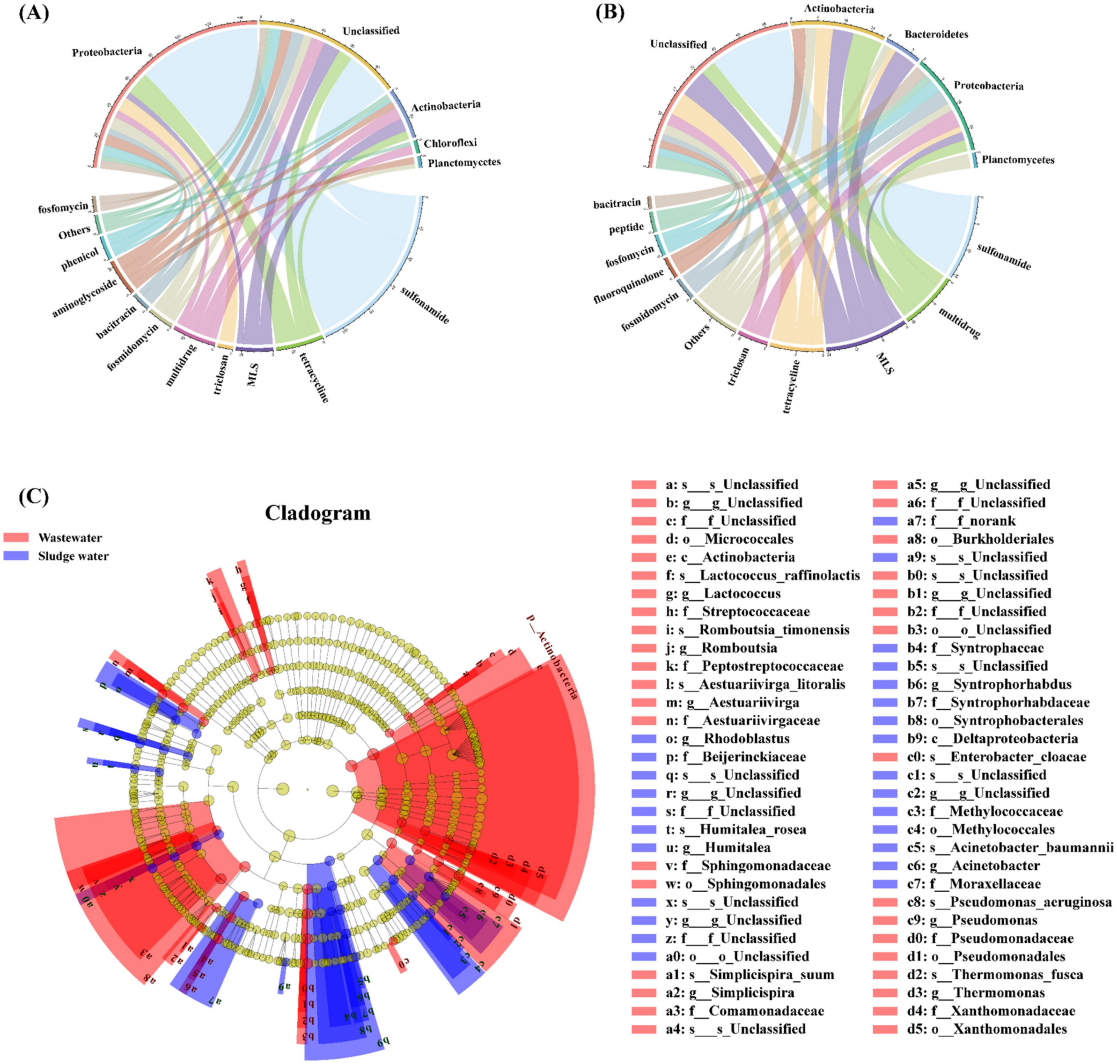
**(A)** Corresponding relationship between ARGs and the host in hospital wastewater. **(B)** Corresponding relationship between ARGs and host in sludge water. **(C)** LEfSe analysis identified the microbial hosts with the greatest difference in different samples.

The relationship between the top 5 hosts by abundance (phylum level) and the top 10 ARG subtypes by abundance is shown in Figures 4A,B. In the hospital wastewater, Proteobacteria dominated in the host, followed by Actinobacteria, Chloroflexi, and Planctomycetes. As shown in Figure 4A, sulfonamide ARGs, triclosan ARGs, fosfomycin ARGs, chloramphenicol ARGs, and fosfomycin ARGs were mainly carried by Proteobacteria; tetracycline ARGs were mainly carried by Proteobacteria and Actinobacteria; macrolide ARGs were mainly carried by Actinobacteria; and multidrug-resistant ARGs co-existed in Proteobacteria, Actinobacteria, and Chloroflexi. Aminoglycoside ARGs co-existed in Proteobacteria, Actinobacteria, and Planctomycetes. Bacitracin ARGs were mainly carried by unclassified bacteria.

The relationship between ARGs and phylum-level hosts in sludge water is shown in Figure 4B. Among the classified bacteria, Actinobacteria and Proteobacteria are both dominant, followed by Bacteroidetes and Planctomycetes. Multidrug-resistant ARGs, tetracycline ARGs, fluoroquinolone ARGs, and bacitracin ARGs are mainly carried by Actinobacteria; and macrolide ARGs are mainly carried by Actinobacteria and Bacteroidetes. Triclosan ARGs, fosfomycin ARGs, and peptide antibiotic ARGs were mainly carried by Proteobacteria. Among them, sulfonamide ARGs were completely carried by unclassified bacteria in sludge water samples, which was quite different from the analysis results in hospital wastewater samples.

Based on the results of ARG host taxonomy annotation, LEfSe was also used for inter-group difference analysis to analyze ARG hosts with significant differences in abundance between groups, and to identify specific ARG hosts that were significantly enriched in different groups (LDA > 2, *p* < 0.05). The different ARG hosts between different sample groups at multiple taxonomic levels are shown in Figure 4C. Actinobacteria is a significantly different species in hospital wastewater, and Deltaproteobacteria is a significantly different species in sludge water.

### 3.3 HPB analysis

**Figure 5:**
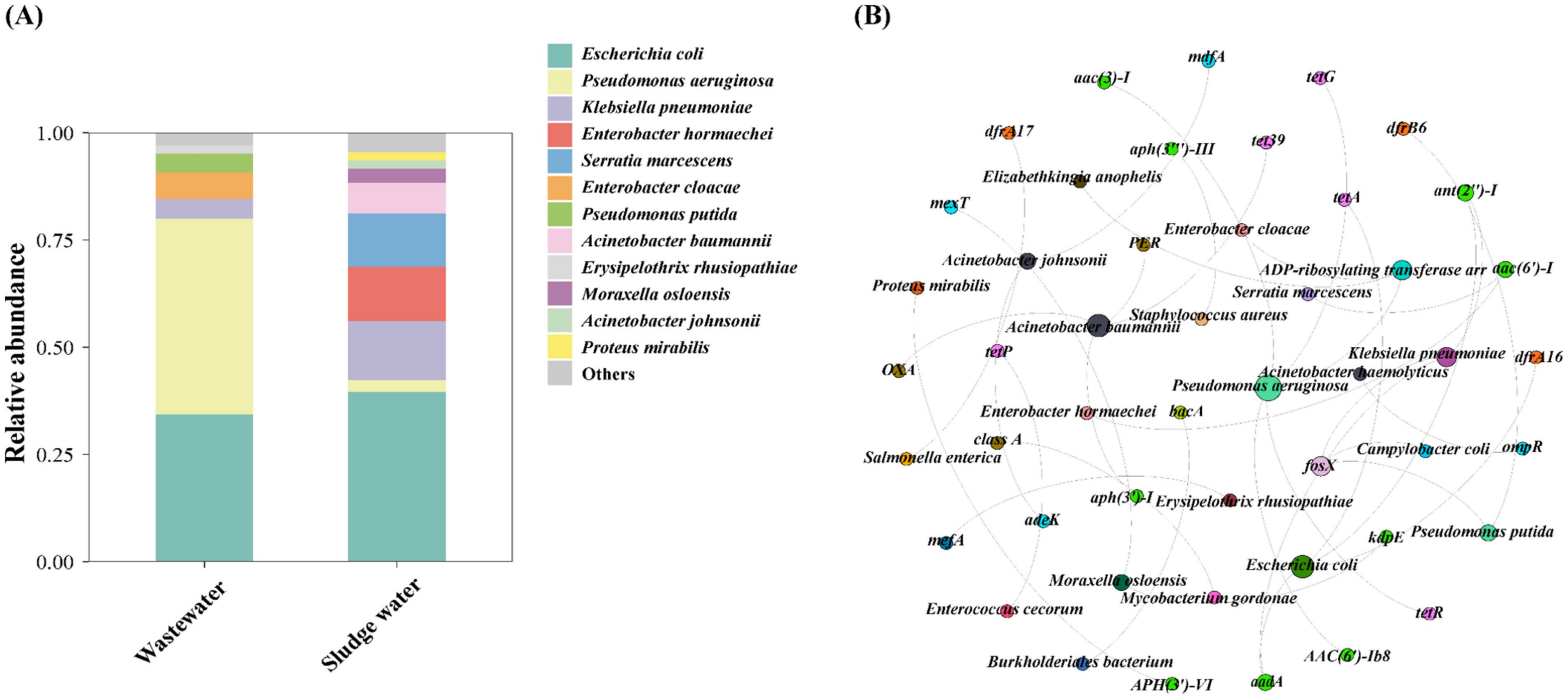
**(A)** Species level HPB host abundance distribution. **(B)** ARG subtypes and HPB host co-occurrence network diagram.

It is shown in Figure 5A that *Escherichia coli* is the main host of HPB in both hospital wastewater and sludge water, and the relative abundance is similar, but the relative abundance of *Pseudomonas aeruginosa* in hospital wastewater is much higher than that in sludge water. At the same time, the relative abundance of *Klebsiella pneumoniae* in different groups was also quite different. In addition, *Enterobacter cloacae*, *Pseudomonas putida,* and *Erysipelothrix rhusiopathiae* were only detected in hospital wastewater. *Enterobacter hormaechei*, *Serratia marcescens*, *Acinetobacter baumannii*, *Moraxella osloensis*, *Acinetobacter johnsonii,* and *Proteus mirabilis* were only detected in sludge water. Based on the network analysis of ARGs and HPB hosts (Figure 5B), *P. aeruginosa*, *E. coli*, *A. baumannii*, and *A. johnsonii* were found to be potential hosts for a variety of ARGs, such as *tetG*, *aac(3)-I*, *tetR*, *OXA*, and *tetA*.

### 3.4 HGT potential analysis

**Figure 6:**
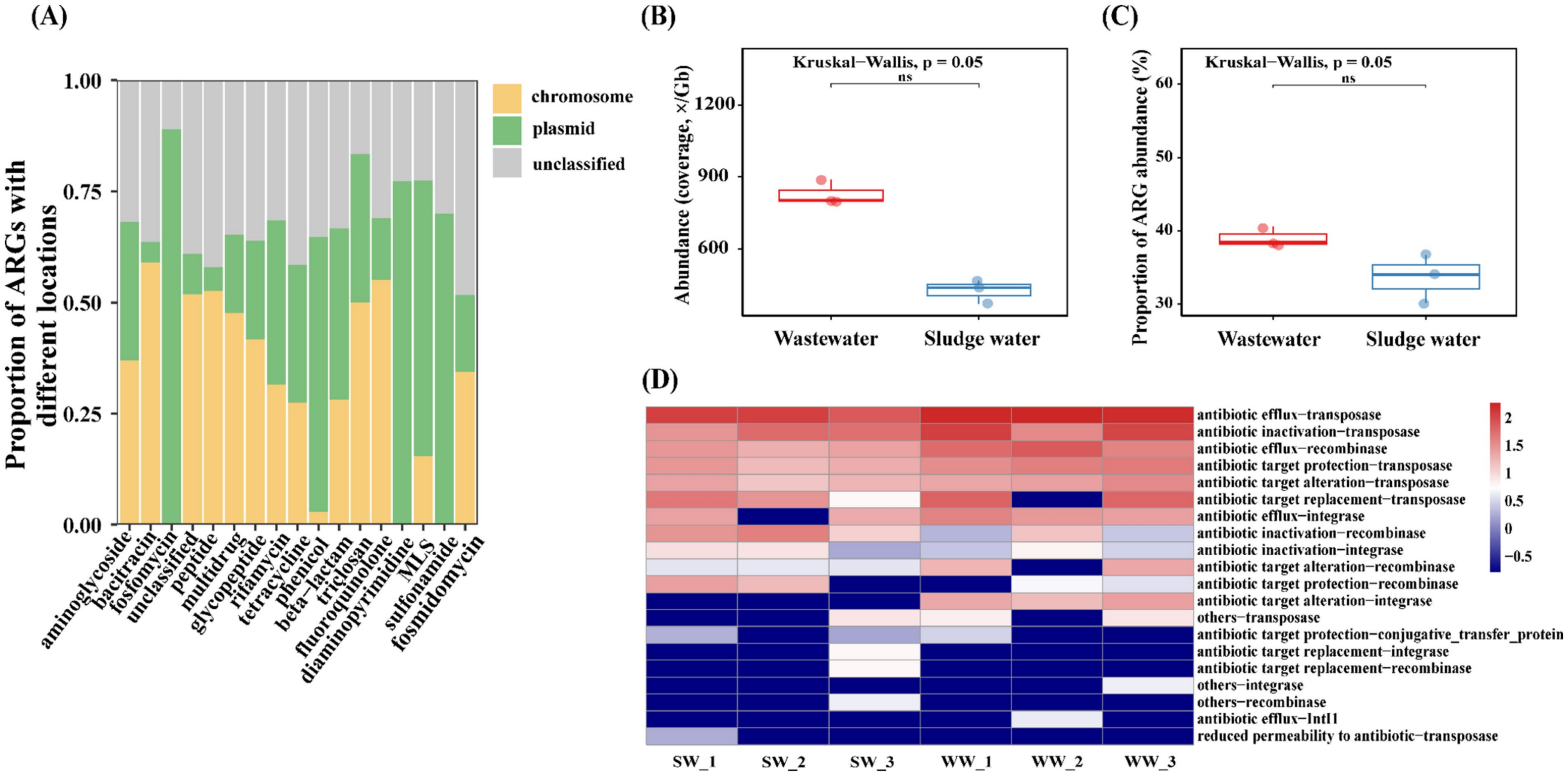
**(A)** The location of the top 17 ARG–MGE co-occurrence ARCs in all groups. **(B)** Abundance of MGEs that co-occur with ARGs. **(C)** Proportion of abundance of ARGs that co-occur with MGEs. **(D)** Co-occurrence of different ARG–MGEs abundance of sexually patterned ARCs in samples.

In this study, the co-occurrence of ARGs with MGEs (ARG–MGE) was analyzed. As shown in Figure 6A, the ARG–MGE co-occurrence ARCs belonging to bacitracin and fluoroquinolone were mainly located on the chromosome, but the ARG–MGE co-occurrence ARCs belonging to sulfonamide, fosfomycin, diaminopyrimidine, and phenicol were mainly located on the plasmid.

The total abundance of MGEs co-occurring with ARGs in hospital wastewater was much higher than that in sludge water (Figure 6B). At the same time, the abundance of ARGs co-occurring with MGEs in hospital wastewater was also higher than that in sludge water (Figure 6C). In addition, based on the different types of ARG–MGE co-occurrences in the sample, this study analyzes the types of ARG–MGE co-occurrence patterns in different sample groups, and the results are shown in Figure 6D. Antibiotic efflux-transposase, antibiotic inactivation-transposase, antibiotic efflux-recombinase, antibiotic target protection-transposase, and antibiotic target alteration-transposase are high-abundance ARG–MGE co-occurrence patterns, which have high consistency in all samples.

**Figure 7:**
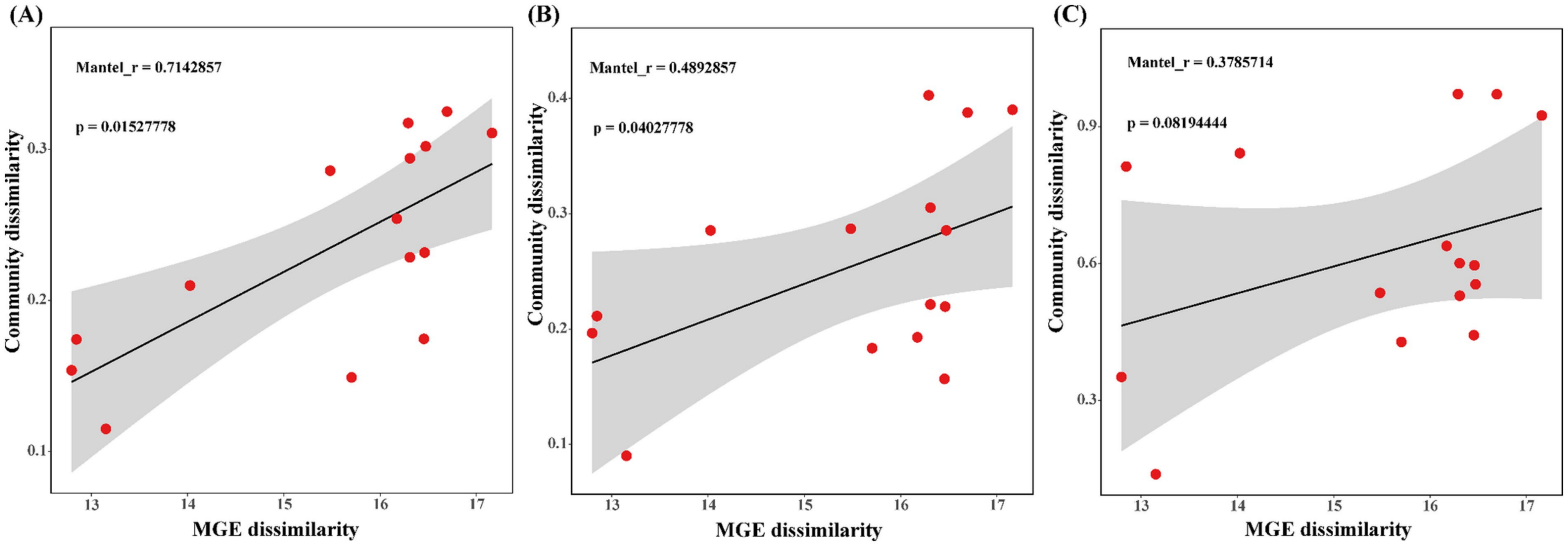
**(A)** Linear regression analysis between ARGs and MGEs community difference distance matrix. **(B)** Linear regression analysis between host and MGEs community difference distance matrix. **(C)** Linear regression analysis between HPB and MGEs community difference distance matrix.

The Bray–Curtis-based Mantel test was used to analyze the correlation between MGE community dissimilarities and those of ARGs, hosts, and HPB. The results, shown in Figure 7, reveal a positive correlation between ARGs, hosts, HPB, and MGEs. Regression analysis showed a significant correlation between the MGE community and the ARG community (Mantel r = 0.71), followed by the host community (Mantel r = 0.49), and the weakest positive correlation with the HPB community (Mantel r = 0.38). Among these, ARGs (*p* = 0.015), hosts (*p* = 0.040), and MGEs were significant, while HPB (*p* = 0.081) and MGEs were not significant.

## 4 Discussion

### 4.1 Enrichment of ARGs in sludge water

The greater the number of ARG types carried by ARCs, the richer the potential antibiotic resistance they provide to the host. Therefore, the proportion of ARCs carrying multiple ARGs in all ARCs and the total abundance of ARGs were counted. The results showed that the total abundance of ARGs and the number of ARCs carrying multiple ARGs in sludge water were lower than those in hospital wastewater (Figure 2A). The reason for this phenomenon is that the activated sludge process can reduce the total abundance of ARGs in sewage (Tong et al., 2019); however, it is worth noting that the activated sludge process can also cause the enrichment of some ARGs while degrading antibiotics (Zhang et al., 2021), thus affecting the abundance distribution of ARGs. Therefore, the abundance of the top 30 ARG subtypes in sludge water is slightly higher than in hospital wastewater (Figures 2C,D). Although the abundance of *β*-lactam ARGs and sulfonamide ARGs is higher, as shown in Figure 2D, the corresponding ARG types, such as *SHV*, *TEM,* and *sul1* are not shown in Figure 2C. This phenomenon may be attributed to the relatively low abundance and high diversity of each ARG subtype corresponding to the two ARG types, enhancing bacterial resistance.

Within the waters of the Tumen River, the major ARGs were multidrug-resistance genes, with significant abundance in resistance genes for multidrug, bacitracin, beta-lactam, macrolide-lincosamide-streptogramin, sulfonamide, fosmidomycin, and polymyxin. Together, these genes accounted for 96.9% of the total ARG abundance, and the ARG diversity showed no obvious ecological boundaries (Zhao et al., 2022). This finding aligns with the major ARGs detected in hospital wastewater and sludge water in this study. The compositional resemblance suggests that hospital wastewater and sludge water significantly influence the presence and distribution of ARGs in the basin, highlighting their potentially strong linkage in the ARG transmission network. The absence of unclear ecological boundaries in ARG diversity within river waters suggests rapid dissemination of ARGs driven by human activities, with the high prevalence of multidrug resistance genes in sludge water potentially spreading throughout the Tumen River Basin, offering localized evidence of this phenomenon. Consequently, targeted interventions in key stages of sludge treatment are crucial to curb ARG accumulation, alongside the establishment of strict discharge standards for sludge water to minimize its environmental effects.

### 4.2 Microbial communities influence ARG transfer and increase pathogenicity

Studies have shown that the microbial community is an important factor in the change of AMR (Li et al., 2015). The bacitracin ARGs in hospital wastewater are mainly carried by unclassified bacteria (Figure 4A), which shows that there may be some unknown bacitracin ARBs in hospital wastewater. In addition, fluoroquinolone antibiotics (such as enrofloxacin and norfloxacin) tend to be enriched in activated sludge (Ouyang et al., 2020), potentially creating environmental pressure that promotes the emergence of corresponding ARGs in sludge water. At the same time, the environmental conditions of activated sludge are quite different from those of hospital wastewater, such as high concentration of organic matter load and oxygen content changes, which lead to the growth and reproduction of certain specific types of microorganisms (Zhang et al., 2021) and become the host of ARGs through HGT. Subsequently, these microorganism enters the sludge water through the sludge concentration, resulting in a different ARGs–host correlation compared to that observed in hospital wastewater.

Furthermore, there is a high abundance of unclassified bacteria in hospital wastewater and sludge water samples, but the reasons for this phenomenon may be different. In hospital wastewater, the large presence of human excreta, combined with high concentrations of antibiotic residues and environmental pollutants, could promote the growth of bacterial communities with strong adaptability and specialized metabolic pathways (Ajala et al., 2022). Activated sludge is a complex community consisting of various microorganisms, many of which may be unclassified or differ from known microbial classifications (Zhang and Zhang, 2023). These microorganisms can be concentrated and transferred into the sludge water during the sludge concentration process.

In this study, there were some differences noted in the correlation between the ARGs and the hosts in hospital wastewater and sludge water samples. Actinobacteria is a significantly different species in hospital wastewater (Figure 4C). Studies have shown that Actinobacteria has a wide range of secondary metabolism, it can produce two-thirds of known antibiotics and has natural antibiotic tolerance (Barka et al., 2016), making it more competitive in hospital wastewater (Xu et al., 2024). At the same time, Deltaproteobacteria is a significantly different species in sludge water (Figure 4C). The results of Sun et al. (2021) showed that Deltaproteobacteria was the main ARB in activated sludge, and the community change of ARB would drive the diversity and abundance distribution of ARGs. Deltaproteobacteria have the ability to degrade complex organic matter and participate in the sulfur cycle (Langwig et al., 2022), which makes them show a significant abundance advantage in sludge water. These findings suggest that addressing the transmission risks of particular bacterial species across different environments and strengthening the surveillance of the microbial community, has an impact on the ARG migration dynamics.

Previous studies focused on the increased priority of ARG-carrying HPB in monitoring and evaluating the risk of antibiotic resistome (Zhu et al., 2023). There are some clinical pathogens and opportunistic pathogens in hospital wastewater, such as *E. coli*, which can cause diarrhea and extraintestinal diseases, and *E. coli* is very likely to transmit ARGs to other enteropathogens (Wang et al., 2018). This also explains why a certain proportion of *Enterobacter hormaechei* appeared in the sludge water. In this study, the proportion of *E. coli* in sludge water (39.6%) was higher than that in hospital wastewater (34.4%), which was consistent with the results of Szekeres et al., that is, the incidence of antibiotic-resistant strains increased after sewage treatment (Farkas et al., 2016; Szekeres et al., 2017).

Globally, ARG-carrying *E. coli* has been extensively found in rivers and coastal surface waters, indicating its robust adaptability and ability to spread ARGs (Blaak et al., 2014; Leonard et al., 2015; Servais and Passerat, 2009). This study found that *E. coli* is the dominant HPB host in sludge water. The high abundance of *E. coli* in sludge water poses a potential risk of transmission to downstream waters or irrigation systems. Moreover, ARG-carrying *E. coli* is highly cytotoxic and may disseminate through bioaerosols, amplifying AMR risks in nearby areas (Wang et al., 2024). HGT between microorganisms in activated sludge has been identified as a key mechanism leading to an increase in the prevalence of antibiotic resistance (Wei et al., 2021). HPB in activated sludge enters sludge water through sludge concentration after acquiring antibiotic resistance, resulting in more types of HPB in sludge water than hospital wastewater (Figure 5A), which further reveals the relevance of AMR between hospital wastewater–activated sludge–sludge water and demonstrates the public health risks of sludge water.

### 4.3 Role of MGEs in the HGT and spread of ARGs

At present, the majority of short-read-based metagenomic studies have identified and quantified the diversity and abundance of ARGs, but they rarely emphasize the specific coexistence structure between ARGs and MGEs, or distinguish their genetic location (plasmids and chromosomes) (Li et al., 2015). In order to further determine the HGT risk of ARGs, this study analyzed the genetic location of ARGs and the co-occurrence of ARG–MGE. The expression level of bacitracin and fluoroquinolone ARG types on chromosomes is higher than that of plasmids (Figure 6A). As the main genetic material of bacteria, chromosomes are subject to strict regulation and stability maintenance mechanism resulting in higher stability. Therefore, ARGs located on chromosomes generally have higher persistence than those on plasmids.

At the same time, plasmids and chromosomes have a tendency to carry different ARG subtypes (Ma et al., 2016; Xiong et al., 2018; Zhao et al., 2020), and fosfomycin ARGs, chloramphenicol ARGs, diaminopyrimidine ARGs, macrolide ARGs, and sulfonamide ARGs tend to complete gene expression in plasmids. As common MGEs, plasmids can replicate and transmit independently of the chromosome of host cells, which may make them more susceptible to environmental conditions, and complete the migration of ARGs through HGT, with strong mobility and a wider host range (Zhao et al., 2020). Notably, certain ARBs can carry multiple plasmids, facilitating the exchange of ARGs among plasmids (Nicolás et al., 2018). Previous studies have shown the high-sequence similarities of plasmid-borne ARGs between clinical and environmental plasmids, which means that ARGs in plasmids are also able to transmit across clinical and environmental boundaries (Wang et al., 2024). Plasmid-mediated ARG migration is dominant in sewage treatment plants (Che et al., 2019). At the same time, sub-inhibitory concentrations of heavy metals can be coupled with plasmids to jointly promote the HGT process of ARGs (Lu et al., 2020). Therefore, these types of ARGs have a high migration risk in hospital wastewater.

In addition, studies have shown that the total abundance of MGEs coexisting with ARGs in the sample group, the abundance information of MGE types, and the proportion of total abundance of ARGs coexisting with MGEs can be used to evaluate the potential mobility of ARGs in different groups (Ke et al., 2023). Our results show that the total abundance of MGEs co-existing with ARGs in hospital wastewater is much higher than that in sludge water. At the same time, the abundance of ARGs co-existing with MGEs in hospital wastewater is also higher than that in sludge water (Figures 6B,C), indicating that hospital wastewater has higher mobility potential than sludge water and tends to complete the transfer through HGT. A variety of antibiotics remain in hospital wastewater, which can be used as a selective pressure to promote the spread of ARGs through horizontal gene transmission (Zhang et al., 2021). Nutrient richness in hospital wastewater, such as organic substances and nitrogen compounds, likely fosters bacterial proliferation and facilitates gene exchange (Szekeres et al., 2017), thereby intensifying ARG–MGE co-occurrence. Conversely, sludge water demonstrates lower abundance of ARGs associated with antibiotic efflux and inactivation, yet it is enriched in ARG–MGE combinations such as antibiotic inactivation-recombinase and antibiotic target protection-recombinase (Figure 6D). This phenomenon may be the result of wastewater treatment processes, which reduce the co-occurrence frequency and diversity of co-occurrence patterns of ARGs and MGEs while concentrating certain ARG–MGE co-occurrences (Xu et al., 2024). Additionally, the microbial community composition formed by wastewater treatment (Cydzik-Kwiatkowska and Zielińska, 2016), potentially influences the abundance of ARG–MGE co-occurrences. Despite sludge water containing fewer MGEs than hospital wastewater overall, both share similar resistance mechanisms and transmission pathways (Figure 6D). Diverse resistance mechanisms enhance the overall antibiotic resistance of the bacterial community, leading to higher AMR risks. The risk of sludge water entering into the environment through agricultural applications, such as sludge fertilization, requires attention, as it signifies the persistent potential for ARGs transmission via sludge water.

Microbial hosts and their coexisting MGEs can affect the distribution and spread of ARGs (Wang et al., 2018). Tracking, managing, and limiting the spread of ARG-associated MGE in pathogenic and symbiotic bacterial species is the key to controlling AMR (Forster et al., 2022). In this study, based on the abundance of ARG subtypes, genus-level host abundance, and species-level HPB abundance, the Mantel test analysis was performed with MGE subtypes abundance to reveal the potential transmission risk of ARGs. In hospital wastewater and sludge water, the difference of ARGs community is the key factor affecting MGEs (Figure 7A). ARGs carried by MGEs are key drivers of the human-mediated spread of AMR (Karkman et al., 2019). The abundant ARGs in hospital wastewater and sludge water mainly complete the potential transmission through coexisting MGEs. These findings provide additional evidence for the pivotal role of MGEs in AMR transmission. Particularly in high-antibiotic-pressure environments, such as hospital wastewater, the activity and abundance of MGEs may significantly increase, thereby accelerating the spread of ARGs (Sun et al., 2023).

Moreover, a moderate positive correlation was observed between variations in host communities and those in MGE communities (Figure 7B). It could result from MGEs affecting bacterial host adaptability (Lopatkin et al., 2016). MGEs can mobilize and integrate in a site-specific or non-specific manner throughout the host genome, accounting for this relationship (Durrant et al., 2020). Nonetheless, host community diversity may also be influenced by broader ecosystem drivers such as environmental conditions and resource availability, leading to a weaker correlation (Zhang et al., 2019). There is no obvious linear correlation between HPB and MGEs (Figure 7C). The reason for this phenomenon may be that HPB has a small proportion in ARG hosts (Wang et al., 2022), and only a minority of HPB associated with identified ARGs, and non-pathogens are the main hosts of ARGs (Xu et al., 2024). At the same time, the abundance and distribution of HPB are primarily influenced by their specific ecological niches and external environmental factors, such as pollutant levels or antibiotic stress (Hocquet et al., 2016), rather than being fully dependent on MGEs. These results indicate that MGEs play an important role in the change of ARGs abundance, and MGEs increase the potential risk of ARGs transferring from hospital wastewater to sludge water and HPB (Fang et al., 2019). Therefore, we should focus on the regulatory mechanism of MGEs on ARG migration in hospital wastewater to further control the risk of environmental resistance. Overall, this study highlights that sludge water, as a potential route for the transmission of ARGs and HPB in hospital wastewater into the environment, poses an alarmingly serious risk, which should be paid more attention to by the government.

## 5 Conclusion

This study highlights the crucial role of sludge water as a pathway for transmitting ARGs and HPB from hospital wastewater into the environment. While activated sludge treatment reduces the overall abundance of ARGs, it also concentrates specific ARG subtypes, increasing their presence in sludge water. The variation in ARG abundance and host patterns between hospital wastewater and sludge water suggests that sludge water is an overlooked reservoir for ARGs and HPB. The co-occurrence of ARG–MGE raises the risk of horizontal gene transfer, further spreading resistance genes into the environment. A large number of ARG subtypes persist through MGEs, intensifying the transmission of antibiotic resistance. This enrichment in sludge water poses serious environmental and public health risks, emphasizing the need for more robust monitoring and regulatory measures. This research underscores the potential of sludge water to contribute to the spread of AMR and calls for strategies to mitigate its impact.

## Data Availability

The datasets presented in this study can be found in online repositories. The names of the repository/repositories and accession number(s) can be found below: https://www.ncbi.nlm.nih.gov/, PRJNA1155112.

## Footnotes

^1^ https://bench.cs.vt.edu/ftp/data/databases/MGEs90.fasta

^2^ https://cloud.majorbio.com

## References

[ref1] Ajala O. J. Tijani J. O. Salau R. B. Abdulkareem A. S. Aremu O. (2022). A review of emerging micro-pollutants in hospital wastewater: environmental fate and remediation options. Results Eng. 16:100671. doi: 10.1016/j.rineng.2022.100671

[ref2] Arango-Argoty G. Garner E. Pruden A. Heath L. S. Vikesland P. Zhang L. (2018). DeepARG: a deep learning approach for predicting antibiotic resistance genes from metagenomic data. Microbiome 6, 1–15. doi: 10.1186/s40168-018-0401-z, PMID: 29391044 PMC5796597

[ref3] Barka E. A. Vatsa P. Sanchez L. Gaveau-Vaillant N. Jacquard C. Klenk H.-P.. (2016). Taxonomy, physiology, and natural products of Actinobacteria. Microbiol. Mol. Biol. Rev. 80, 1–43. doi: 10.1128/MMBR.00019-15, PMID: 26609051 PMC4711186

[ref4] Blaak H. de Kruijf P. Hamidjaja R. A. van Hoek A. H. de Roda Husman A. M. Schets F. M. (2014). Prevalence and characteristics of ESBL-producing *E. coli* in Dutch recreational waters influenced by wastewater treatment plants. Vet. Microbiol. 171, 448–459. doi: 10.1016/j.vetmic.2014.03.007, PMID: 24690376

[ref5] Buchfink B. Xie C. Huson D. H. (2015). Fast and sensitive protein alignment using DIAMOND. Nat. Methods 12, 59–60. doi: 10.1038/nmeth.3176, PMID: 25402007

[ref6] Cai L. Sun J. Yao F. Yuan Y. Zeng M. Zhang Q.. (2021). Antimicrobial resistance bacteria and genes detected in hospital sewage provide valuable information in predicting clinical antimicrobial resistance. Sci. Total Environ. 795:148815. doi: 10.1016/j.scitotenv.2021.148815, PMID: 34247085

[ref7] Che Y. Xia Y. Liu L. Li A.-D. Yang Y. Zhang T. (2019). Mobile antibiotic resistome in wastewater treatment plants revealed by Nanopore metagenomic sequencing. Microbiome 7, 1–13. doi: 10.1186/s40168-019-0663-0, PMID: 30898140 PMC6429696

[ref8] Chen J. Wang T. Zhang K. Luo H. Chen W. Mo Y.. (2021). The fate of antibiotic resistance genes (ARGs) and mobile genetic elements (MGEs) from livestock wastewater (dominated by quinolone antibiotics) treated by microbial fuel cell (MFC). Ecotoxicol. Environ. Saf. 218:112267. doi: 10.1016/j.ecoenv.2021.112267, PMID: 33932652

[ref9] Chen S. Zhou Y. Chen Y. Gu J. (2018). Fastp: an ultra-fast all-in-one FASTQ preprocessor. Bioinformatics 34, i884–i890. doi: 10.1093/bioinformatics/bty560, PMID: 30423086 PMC6129281

[ref10] Cydzik-Kwiatkowska A. Zielińska M. (2016). Bacterial communities in full-scale wastewater treatment systems. World J. Microbiol. Biotechnol. 32, 1–8. doi: 10.1007/s11274-016-2012-9, PMID: 26931606 PMC4773473

[ref11] Durrant M. G. Li M. M. Siranosian B. A. Montgomery S. B. Bhatt A. S. (2020). A bioinformatic analysis of integrative mobile genetic elements highlights their role in bacterial adaptation. Cell Host Microbe 27, 140–153.e9. doi: 10.1016/j.chom.2019.10.022, PMID: 31862382 PMC6952549

[ref12] Erdincler A. Vesilind P. (2003). Effect of sludge water distribution on the liquid–solid separation of a biological sludge. J. Environ. Sci. Health A 38, 2391–2400. doi: 10.1081/ESE-120023439, PMID: 14524691

[ref13] Fang P. Peng F. Gao X. Xiao P. Yang J. (2019). Decoupling the dynamics of bacterial taxonomy and antibiotic resistance function in a subtropical urban reservoir as revealed by high-frequency sampling. Front. Microbiol. 10:1448. doi: 10.3389/fmicb.2019.01448, PMID: 31312186 PMC6614491

[ref14] Farkas A. Bocoş B. Butiuc-Keul A. (2016). Antibiotic resistance and intI1 carriage in waterborne Enterobacteriaceae. Water Air Soil Pollut. 227, 1–11. doi: 10.1007/s11270-016-2944-6, PMID: 39850651

[ref15] Forster S. C. Liu J. Kumar N. Gulliver E. L. Gould J. A. Escobar-Zepeda A.. (2022). Strain-level characterization of broad host range mobile genetic elements transferring antibiotic resistance from the human microbiome. Nat. Commun. 13:1445. doi: 10.1038/s41467-022-29096-9, PMID: 35301310 PMC8931123

[ref16] He P. Zhou Y. Shao L. Huang J. Yang Z. Lü F. (2019). The discrepant mobility of antibiotic resistant genes: evidence from their spatial distribution in sewage sludge flocs. Sci. Total Environ. 697:134176. doi: 10.1016/j.scitotenv.2019.134176, PMID: 31491625

[ref17] Hocquet D. Muller A. Bertrand X. (2016). What happens in hospitals does not stay in hospitals: antibiotic-resistant bacteria in hospital wastewater systems. J. Hosp. Infect. 93, 395–402. doi: 10.1016/j.jhin.2016.01.010, PMID: 26944903

[ref18] Hutinel M. Larsson D. J. Flach C.-F. (2022). Antibiotic resistance genes of emerging concern in municipal and hospital wastewater from a major Swedish city. Sci. Total Environ. 812:151433. doi: 10.1016/j.scitotenv.2021.151433, PMID: 34748849

[ref19] Kaliakatsos A. Gounaki I. Dokianakis S. Maragkaki E. Stasinakis A. S. Gyparakis S.. (2024). Treatment of hospital wastewater: emphasis on ecotoxicity and antibiotic resistance genes. J. Chem. Technol. Biotechnol. 99, 2129–2138. doi: 10.1002/jctb.7329

[ref20] Karkman A. Pärnänen K. Larsson D. J. (2019). Fecal pollution can explain antibiotic resistance gene abundances in anthropogenically impacted environments. Nat. Commun. 10:80. doi: 10.1038/s41467-018-07992-3, PMID: 30622259 PMC6325112

[ref21] Ke Y. Sun W. Jing Z. Zhu Y. Zhao Z. Xie S. (2023). Antibiotic resistome alteration along a full-scale drinking water supply system deciphered by metagenome assembly: regulated by seasonality, mobile gene elements and antibiotic resistant gene hosts. Sci. Total Environ. 862:160887. doi: 10.1016/j.scitotenv.2022.160887, PMID: 36521611

[ref22] Khan F. A. Söderquist B. Jass J. (2019). Prevalence and diversity of antibiotic resistance genes in Swedish aquatic environments impacted by household and hospital wastewater. Front. Microbiol. 10:688. doi: 10.3389/fmicb.2019.00688, PMID: 31019498 PMC6458280

[ref23] Krawczyk P. S. Lipinski L. Dziembowski A. (2018). PlasFlow: predicting plasmid sequences in metagenomic data using genome signatures. Nucleic Acids Res. 46:e35. doi: 10.1093/nar/gkx1321, PMID: 29346586 PMC5887522

[ref24] Langwig M. V. de V. Dombrowski N. Seitz K. W. Rambo I. M. Greening C.. (2022). Large-scale protein level comparison of Deltaproteobacteria reveals cohesive metabolic groups. ISME J. 16, 307–320. doi: 10.1038/s41396-021-01057-y, PMID: 34331018 PMC8692467

[ref25] Leonard A. F. Zhang L. Balfour A. J. Garside R. Gaze W. H. (2015). Human recreational exposure to antibiotic resistant bacteria in coastal bathing waters. Environ. Int. 82, 92–100. doi: 10.1016/j.envint.2015.02.013, PMID: 25832996

[ref26] Li A.-D. Li L.-G. Zhang T. (2015). Exploring antibiotic resistance genes and metal resistance genes in plasmid metagenomes from wastewater treatment plants. Front. Microbiol. 6:1025. doi: 10.3389/fmicb.2015.01025 26441947 PMC4585309

[ref27] Li C. Lu J. Liu J. Zhang G. Tong Y. Ma N. (2016). Exploring the correlations between antibiotics and antibiotic resistance genes in the wastewater treatment plants of hospitals in Xinjiang, China. Environ. Sci. Pollut. Res. 23, 15111–15121. doi: 10.1007/s11356-016-6688-z, PMID: 27094265

[ref28] Lopatkin A. J. Sysoeva T. A. You L. (2016). Dissecting the effects of antibiotics on horizontal gene transfer: analysis suggests a critical role of selection dynamics. BioEssays 38, 1283–1292. doi: 10.1002/bies.201600133, PMID: 27699821 PMC6541220

[ref29] Lu J. Wang Y. Jin M. Yuan Z. Bond P. Guo J. (2020). Both silver ions and silver nanoparticles facilitate the horizontal transfer of plasmid-mediated antibiotic resistance genes. Water Res. 169:115229. doi: 10.1016/j.watres.2019.115229, PMID: 31783256

[ref30] Ma L. Li B. Jiang X.-T. Wang Y.-L. Xia Y. Li A.-D.. (2017). Catalogue of antibiotic resistome and host-tracking in drinking water deciphered by a large scale survey. Microbiome 5, 1–12. doi: 10.1186/s40168-017-0369-0, PMID: 29179769 PMC5704573

[ref31] Ma L. Xia Y. Li B. Yang Y. Li L.-G. Tiedje J. M.. (2016). Metagenomic assembly reveals hosts of antibiotic resistance genes and the shared resistome in pig, chicken, and human feces. Environ. Sci. Technol. 50, 420–427. doi: 10.1021/acs.est.5b03522, PMID: 26650334

[ref32] Majlander J. Anttila V.-J. Nurmi W. Seppälä A. Tiedje J. Muziasari W. (2021). Routine wastewater-based monitoring of antibiotic resistance in two Finnish hospitals: focus on carbapenem resistance genes and genes associated with bacteria causing hospital-acquired infections. J. Hosp. Infect. 117, 157–164. doi: 10.1016/j.jhin.2021.09.008, PMID: 34537275

[ref33] Martínez J. L. Coque T. M. Baquero F. (2015). What is a resistance gene? Ranking risk in resistomes. Nat. Rev. Microbiol. 13, 116–123. doi: 10.1038/nrmicro3399, PMID: 25534811

[ref34] Nicolás M. F. Ramos P. I. P. Marques de Carvalho F. Camargo D. R. de Fátima Morais Alves C. Loss de Morais G.. (2018). Comparative genomic analysis of a clinical isolate of Klebsiella quasipneumoniae subsp. similipneumoniae, a KPC-2 and OKP-B-6 beta-lactamases producer harboring two drug-resistance plasmids from Southeast Brazil. Front. Microbiol. 9:220. doi: 10.3389/fmicb.2018.00220, PMID: 29503635 PMC5820359

[ref35] Ouyang J. Li C. Wei L. Wei D. Zhao M. Zhao Z.. (2020). Activated sludge and other aerobic suspended culture processes. Water Environ. Res. 92, 1717–1725. doi: 10.1002/wer.1427, PMID: 32762078

[ref36] Perry M. R. Lepper H. C. McNally L. Wee B. A. Munk P. Warr A.. (2021). Secrets of the hospital underbelly: patterns of abundance of antimicrobial resistance genes in hospital wastewater vary by specific antimicrobial and bacterial family. Front. Microbiol. 12:703560. doi: 10.3389/fmicb.2021.703560, PMID: 34566912 PMC8461093

[ref37] Pires D. de Kraker M. E. A. Tartari E. Abbas M. Pittet D. (2017). “Fight antibiotic resistance—It’s in your hands”: call from the World Health Organization for 5th may 2017. Clin. Infect. Dis. 64, 1780–1783. doi: 10.1093/cid/cix226 28487167

[ref38] Servais P. Passerat J. (2009). Antimicrobial resistance of fecal bacteria in waters of the seine river watershed (France). Sci. Total Environ. 408, 365–372. doi: 10.1016/j.scitotenv.2009.09.042, PMID: 19853889

[ref39] Sun F. Xu Z. Fan L. (2021). Response of heavy metal and antibiotic resistance genes and related microorganisms to different heavy metals in activated sludge. J. Environ. Manag. 300:113754. doi: 10.1016/j.jenvman.2021.113754, PMID: 34543965

[ref40] Sun J. Yuan Y. Cai L. Zeng M. Li X. Yao F.. (2023). Metagenomic evidence for antibiotics-driven co-evolution of microbial community, resistome and mobilome in hospital sewage. Environ. Pollut. 327:121539. doi: 10.1016/j.envpol.2023.121539, PMID: 37019259

[ref41] Szekeres E. Baricz A. Chiriac C. M. Farkas A. Opris O. Soran M.-L.. (2017). Abundance of antibiotics, antibiotic resistance genes and bacterial community composition in wastewater effluents from different Romanian hospitals. Environ. Pollut. 225, 304–315. doi: 10.1016/j.envpol.2017.01.054, PMID: 28347610

[ref42] Tong J. Tang A. Wang H. Liu X. Huang Z. Wang Z.. (2019). Microbial community evolution and fate of antibiotic resistance genes along six different full-scale municipal wastewater treatment processes. Bioresour. Technol. 272, 489–500. doi: 10.1016/j.biortech.2018.10.079, PMID: 30391842

[ref43] Vaxelaire J. Cézac P. (2004). Moisture distribution in activated sludges: a review. Water Res. 38, 2215–2230. doi: 10.1016/j.watres.2004.02.021, PMID: 15142782

[ref44] Wang J. Pan R. Dong P. Liu S. Chen Q. Borthwick A. G. L.. (2022). Supercarriers of antibiotic resistome in a world's large river. Microbiome 10:111. doi: 10.1186/s40168-022-01294-z, PMID: 35897057 PMC9331799

[ref45] Wang Q. Wang P. Yang Q. (2018). Occurrence and diversity of antibiotic resistance in untreated hospital wastewater. Sci. Total Environ. 621, 990–999. doi: 10.1016/j.scitotenv.2017.10.128, PMID: 29054666

[ref46] Wang Y. Yang K. Li L. Yang L. Zhang S. Yu F.. (2024). Change characteristics, bacteria host, and spread risks of bioaerosol ARGs/MGEs from different stages in sewage and sludge treatment process. J. Hazard. Mater. 469:134011. doi: 10.1016/j.jhazmat.2024.134011, PMID: 38492400

[ref47] Wang X. Zhang H. Yu S. Li D. Gillings M. R. Ren H.. (2024). Inter-plasmid transfer of antibiotic resistance genes accelerates antibiotic resistance in bacterial pathogens. ISME J. 18:wrad032. doi: 10.1093/ismejo/wrad032, PMID: 38366209 PMC10881300

[ref48] Wei Z. Feng K. Wang Z. Zhang Y. Yang M. Zhu Y.-G.. (2021). High-throughput single-cell technology reveals the contribution of horizontal gene transfer to typical antibiotic resistance gene dissemination in wastewater treatment plants. Environ. Sci. Technol. 55, 11824–11834. doi: 10.1021/acs.est.1c01250, PMID: 34415164

[ref49] Xiong W. Wang Y. Sun Y. Ma L. Zeng Q. Jiang X.. (2018). Antibiotic-mediated changes in the fecal microbiome of broiler chickens define the incidence of antibiotic resistance genes. Microbiome 6, 1–11. doi: 10.1186/s40168-018-0419-2, PMID: 29439741 PMC5811963

[ref50] Xu C. Hu C. Li F. Liu W. Xu Y. Shi D. (2024). Antibiotic resistance genes risks in relation to host pathogenicity and mobility in a typical hospital wastewater treatment process. Environ. Res. 259:119554. doi: 10.1016/j.envres.2024.119554, PMID: 38964571

[ref51] Yi X. Liang J.-L. Su J.-Q. Jia P. Lu J.-l. Zheng J.. (2022). Globally distributed mining-impacted environments are underexplored hotspots of multidrug resistance genes. ISME J. 16, 2099–2113. doi: 10.1038/s41396-022-01258-z, PMID: 35688988 PMC9381775

[ref52] Yin S. Gao L. Fan X. Gao S. Zhou X. Jin W.. (2024). Performance of sewage sludge treatment for the removal of antibiotic resistance genes: status and prospects. Sci. Total Environ. 907:167862. doi: 10.1016/j.scitotenv.2023.167862, PMID: 37865259

[ref53] Zainab S. M. Junaid M. Xu N. Malik R. N. (2020). Antibiotics and antibiotic resistant genes (ARGs) in groundwater: a global review on dissemination, sources, interactions, environmental and human health risks. Water Res. 187:116455. doi: 10.1016/j.watres.2020.116455, PMID: 33032106

[ref54] Zhang L. Shen Z. Fang W. Gao G. (2019). Composition of bacterial communities in municipal wastewater treatment plant. Sci. Total Environ. 689, 1181–1191. doi: 10.1016/j.scitotenv.2019.06.432 31466158

[ref55] Zhang Y. Zhang T. (2023). Culturing the uncultured microbial majority in activated sludge: a critical review. Crit. Rev. Environ. Sci. Technol. 53, 601–624. doi: 10.1080/10643389.2022.2077063

[ref56] Zhang H. Zhang Z. Song J. Cai L. Yu Y. Fang H. (2021). Foam shares antibiotic resistomes and bacterial pathogens with activated sludge in wastewater treatment plants. J. Hazard. Mater. 408:124855. doi: 10.1016/j.jhazmat.2020.124855, PMID: 33373956

[ref57] Zhang C. Zhao Z. Dong S. Zhou D. (2021). Simultaneous elimination of amoxicillin and antibiotic resistance genes in activated sludge process: contributions of easy-to-biodegrade food. Sci. Total Environ. 764:142907. doi: 10.1016/j.scitotenv.2020.142907, PMID: 33757248

[ref58] Zhao C. Li C. Wang X. Cao Z. Gao C. Su S.. (2022). Monitoring and evaluation of antibiotic resistance genes in three rivers in Northeast China. Environ. Sci. Pollut. Res. 29, 44148–44161. doi: 10.1007/s11356-022-18555-x, PMID: 35122641

[ref59] Zhao R. Yu K. Zhang J. Zhang G. Huang J. Ma L.. (2020). Deciphering the mobility and bacterial hosts of antibiotic resistance genes under antibiotic selection pressure by metagenomic assembly and binning approaches. Water Res. 186:116318. doi: 10.1016/j.watres.2020.116318, PMID: 32871290

[ref60] Zhu L. Yuan L. Shuai X.-Y. Lin Z.-J. Sun Y.-J. Zhou Z.-C.. (2023). Deciphering basic and key traits of antibiotic resistome in influent and effluent of hospital wastewater treatment systems. Water Res. 231:119614. doi: 10.1016/j.watres.2023.119614, PMID: 36682238

